# Psychostimulant Medications for Physical Function and Spasticity in Children With Cerebral Palsy: Protocol for a Randomized Controlled Trial

**DOI:** 10.2196/53728

**Published:** 2024-03-05

**Authors:** Mansour Alotaibi, Anwar B Almutairi, Saleh Alhirsan, Afrah Alkazemi, Maha Alharbi, Naif Alrashdi, Ahmad Taqi, Bibi Alamiri, Laura Vogtle, Mohammed M Alqahtani

**Affiliations:** 1 Department of Rehabilitation Faculty of Applied Medical Sciences Northern Border University Arar Saudi Arabia; 2 Center for Health Research Northern Border University Arar Saudi Arabia; 3 Department of Physical Therapy Faculty of Allied Health Kuwait University Kuwait City Kuwait; 4 Department of Physical Therapy College of Applied Medical Sciences Jouf University Aljouf Saudi Arabia; 5 Department of Pharmacy Practice College of Pharmacy Kuwait University Kuwait City Kuwait; 6 Department of Physical Therapy and Health Rehabilitation College of Applied Medical Sciences Majmaah University Al-Majmaah Saudi Arabia; 7 The Health and Scientific Research Center Majmaah University Majmaah Saudi Arabia; 8 Kuwait Center for Mental Health Public Authority for Disability Affairs Almanara Kuwait; 9 Department of Occupational Therapy School of Health Professions University of Alabama at Birmingham Birmingham, AL United States; 10 Department of Respiratory Therapy College of Applied Medical Sciences King Saud bin Abdulaziz University for Health Sciences Riyadh Saudi Arabia; 11 King Abdullah International Medical Research Center Riyadh Saudi Arabia

**Keywords:** cerebral palsy, CNS stimulants, spasticity, motor performance, gross motor function, psychostimulant, medications, physical function, CP, children, child, pediatrics, pediatric, impairment, movement, central nervous system, safety, tolerability, efficacy, methylphenidate, modafinil, Kuwait, rehabilitation, physical therapy

## Abstract

**Background:**

Cerebral palsy (CP) is a prevalent nonprogressive disorder that leads to impaired movement (ie, spasticity), posture, and balance, which affects functions such as walking and upper extremity tasks. Current medical treatments show efficacy in improving motor performance but have considerable side effects. Emerging off-label use of central nervous system (CNS) medications for improving motor performance has shown promising results in children with CP and other populations.

**Objective:**

The aim of this study is to describe a protocol for a pilot randomized controlled trial (RCT) to examine the safety, tolerability, and efficacy of methylphenidate (MPH) and modafinil on spasticity and motor performance in children with CP.

**Methods:**

This will be a protocol study for a pilot, triple-masked, placebo-controlled RCT (a class I trial following the American Academy of Neurology criteria) with blinded patients, outcome assessors, and intervention delivery team. Eligible children should be diagnosed with CP levels I or II based on the Gross Motor Function Classification System and be aged between 7 and 12 years. Thirty-six children with CP will be randomized into 3 groups to receive (1) MPH (2.5 mg of MPH + 100 mg placebo), (2) modafinil (100 mg modafinil + 2.5 mg placebo), or (3) a placebo (2.5 mg placebo + 100 mg placebo), in addition to physical therapy for 12 weeks. Primary outcomes include the Gross Motor Function Measure–66 and the Modified Ashworth Scale. Secondary outcomes include the Timed Up and Go test, 5 Time Sit to Stand test, Modified Clinical Test for Sensory Interaction of Balance, and 10-Meter Walk Test.

**Results:**

The protocol has been accepted by Kuwait University (VDR/EC-225) and the Ministry of Health of Kuwait (2022/2157). The inclusion of participants will start in June 2024.

**Conclusions:**

The combination of CNS stimulant medications and controlling for rehabilitation has not been studied yet. The findings of this study may determine if using CNS stimulant medications is beneficial for the reduction of spasticity and improvement of physical function in children with spastic CP.

**Trial Registration:**

ClinicalTrials.gov NCT05675098; https://clinicaltrials.gov/study/NCT05675098

**International Registered Report Identifier (IRRID):**

PRR1-10.2196/53728

## Introduction

### Background

Cerebral palsy (CP) is a group of disorders that cause permanent, nonprogressive damage to the developing brain [1], leading to impairments in movement and posture, as well as balance deficits [2,3]. These impairments could affect gross motor skills [4], such as gait [5], upper limb tasks (eg, reaching) [6], and oral motor function (eg, eating and swallowing) [7], which could contribute to limitations across a variety of life domains, including self-care, education and work, and recreational activities [8].

CP is one of the leading causes of disability [2], affecting about 1 in 500 children, with estimated prevalence of 17 million individuals globally [9]. CP can be classified into different categories based on motor impairments, including spasticity (increased muscle excitability) [10], dyskinesia (uncontrollable random movement) [11], ataxia (impaired coordination) [12], or mixed movement disorders [3].

Spastic disorders are the most common type of CP that affects motor activities essential for activities of daily living (ADL) [3]. Treatment of CP spastic disorders includes pharmacological medications (oral and injected), surgical therapy, and nonpharmacological therapy (ie, rehabilitation or constraint-induced movement therapy [13,14]). Pharmacological medications with different mechanisms of action can be used to reduce spasticity in children with CP. For instance, using botulinum toxin type A (BoNT-A) injections is efficacious in reducing spasticity of the upper extremities (UEs) [15] and lower extremities (LEs) [16], which helps in improving the overall motor function in children with CP [17]. BoNT-A inhibits acetylcholine production from the presynaptic terminal, causing a decrease in muscle excitability [17]. Based on the current CP clinical practice guidelines, BoNT-A is considered a safe and effective intervention for spasticity in children and adolescents with CP [18-21]. However, BoNT-A effects are peripheral, targeting specific muscles, and have a temporary influence on spasticity [15,16]. Importantly, BoNT-A has been criticized due to its potential to induce muscle weakness in a condition that is characterized by motor impairments [17].

Other pharmacological medications can reduce spasticity in children with CP, such as baclofen (tablets or intrathecal injections) and diazepam (tablets) [20]. These medications reduce spasticity by improving the affinity of gamma-amino butyric acid (GABA) on its receptors, which in turn blocks excitatory neurotransmitters [22,23]. Notably, these medications have shown effectiveness in both reducing spasticity and improving motor performance in children with CP [24,25]. Nevertheless, baclofen and diazepam have potential side effects that could impact their use, including drowsiness and potential muscle weakness [20]. These medications counter a key factor in improving motor performance by decreasing the rate of motor firing [20]. Therefore, there is a critical need to explore the effects of medications that could both improve motor performance and reduce spasticity but also exhibit minimal side effects in children with CP.

In the past 2 decades, central nervous system (CNS) stimulants such as methylphenidate (MPH) have been reported to improve motor performance when used in children with attention-deficit/hyperactivity disorder (ADHD) [26,27]. Furthermore, a case report documented that a woman aged 44 years with CP (mixed type—choreoathetosis with spasticity) consumed another CNS stimulant (amphetamine) recreationally and observed a remarkable reduction in her spasticity, which encouraged her physician to prescribe MPH for spasticity reduction [28]. This woman had a considerable long-term decline in spasticity and choreoathetosis with MPH use [28]. Another CNS stimulant (modafinil) showed improvements in gait and spasticity among children with CP [29-31]. Nevertheless, the effects of modafinil on spasticity and motor improvement have been inconsistent [32]. Consequently, potential CNS stimulants such as MPH and modafinil are hypothesized to reduce spasticity and improve motor performance in children with CP due to alteration in neurochemicals in the brain (ie, changes in neuroplasticity) [33].

Rehabilitation interventions for children with CP produce changes in neuroplasticity [34], which could lead to long-term improvements in motor function. Specifically, early interventions for spasticity and motor function may be associated with improved motor performance due to enhancement of sensory feedback plasticity [35]. Sensory feedback is an essential modifiable factor for motor learning by iteration, which alters connections between primary motor and somatosensory cortices [36,37]. Therefore, the use of CNS stimulants, in addition to rehabilitation interventions, could offer long-term improvements in spasticity and motor performance in children with CP. Using CNS stimulants may offer advantages over existing pharmacological treatments, primarily because they do not induce muscle weakness [17]. Given the limited studies that have examined the effects of CNS stimulants on motor performance, with or without rehabilitation, in children with CP, a pilot experimental research study is warranted.

### Objective

We designed a triple-masked pilot randomized controlled trial (RCT) that aims to examine the safety and tolerability of MPH and modafinil (aim 1) as well as to assess trends in changes in gross motor function and spasticity with the use of MPH and modafinil in children with CP (aim 2). In this placebo-controlled study, participants will be randomized into 3 groups that will receive MPH, modafinil, or placebo tablets. We hypothesize that using MPH or modafinil will be tolerable and safe for children with CP. We also hypothesize that there will be trends toward improvement in gross motor function and spasticity in children with CP.

## Methods

### Ethical Considerations

Ethical approval for this study has been obtained from Kuwait University (VDR/EC-225) and the Ministry of Health of Kuwait (2022/2157). Prior to initiation of this study, participants and their legal guardians will be informed about the study and written consent and assent will be obtained from each participant. We followed the SPIRIT (Standard Protocol Items: Recommendation for Interventional Trials) guidelines for the preparation of this protocol [38]. The SPIRIT guidelines are a 33-item checklist that allows researchers to draft and plan high-quality RCTs and describe elements of the content of the RCT. The protocol of this study has been registered at ClinicalTrials.gov (NCT05675098).

### Study Design

**Figure 1:**
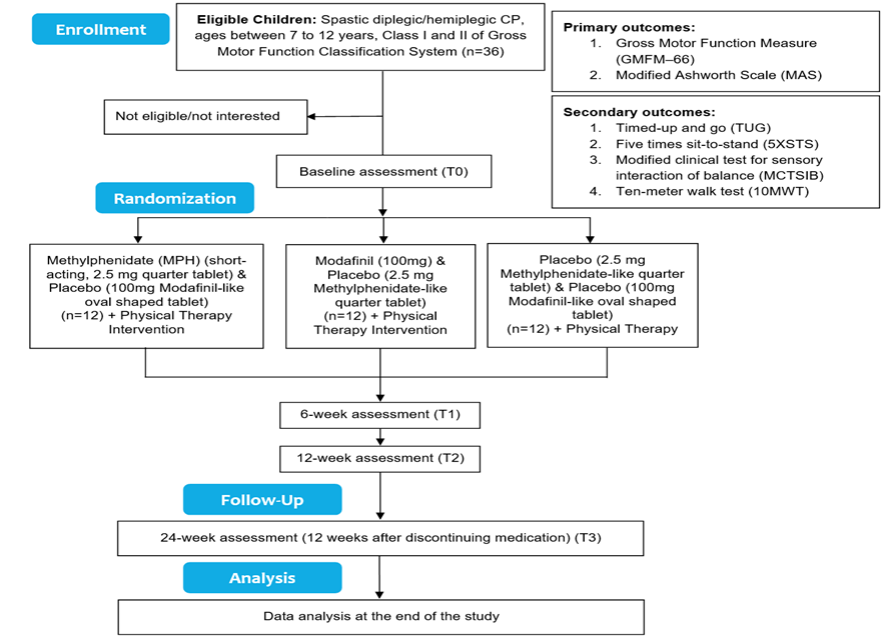
Consolidated Standards of Reporting Trials (CONSORT) flowchart of the proposed randomized controlled trial.

This study will be a single-center, prospective, triple-masked, placebo-controlled pilot RCT. The study design is informed by the American Academy of Neurology (AAN) criteria for evidence classification as a class I study [39]. To meet these criteria, this study will (1) be triple-masked (blinded) to minimize the risk of bias (ie, patients and legal guardians, treatment providers, and outcome assessors will be blinded), (2) use the intent-to-treat principle to account for dropouts, (3) have only 2 primary outcomes (the Gross Motor Function Measure [GMFM] and Modified Ashworth Scale [MAS]), (4) have a prespecified minimal clinical detectable change in the primary outcomes, and (5) use a powerful method to correct for multiple comparisons for secondary outcomes. This study will also use a placebo-control group as an active control; this is due to the fact that there are no medications that possess CNS-stimulant qualities that have been tested for the purpose of the study. This study will examine the effects of a 12-week administration of MPH, modafinil, or placebo with the GMFM and MAS, with a testing session in the middle of the study period (ie, at the 6-week mark). This is integral, as it will allow for monitoring changes in the primary outcomes at multiple time points. The study will also examine the long-lasting effects of these medications (MPH and modafinil) at 12 weeks after the conclusion of the intervention (ie, at 24 weeks) to assess if any detected trends of improvement last beyond the period of the intervention. This study will be guided by the CONSORT (Consolidated Standards of Reporting Trials) guidelines for recruitment (Figure 1).

### Recruitment

#### Participants

Formal power calculation will not be carried out because this is a pilot study. Children with CP (N=36) aged 7 to 12 years will be enrolled (ie, 12 children per group). Children will be recruited from hospitals around Kuwait using several advertisement methods, including flyers, email notifications to health care providers, and social media advertisements (eg, on X, formerly known as Twitter). Additionally, we will use the snowball recruitment method for this study. The study will be conducted at Jaber Al-Ahmad Hospital, Kuwait.

#### Inclusion Criteria

Children will be screened and considered for eligibility if they are diagnosed with spastic diplegic or hemiplegic CP by a pediatric neurologist with at least 5 years of experience with this population, aged 7 to 2 years, male or female, classified as level I or II on the Gross Motor Function Classification System (GMFCS), (ie, they are able to walk independently with or without limitation) [40], and have received physical therapy for at least 3 months. GMFCS level will be determined by the treating therapist of the participant according to the expanded and revised version of the GMFCS [41]. If eligible, participants will only follow the rehabilitation intervention described in this protocol. Medicine management will continue as prescribed by the treating physician.

#### Exclusion Criteria

Children will be excluded if they have had a seizure in the past 6 months, have been diagnosed with ADHD, have had any surgery within the last 6 months, use medications that interfere with spasticity (eg, baclofen), are unable to follow simple commands, or have LE (hip, knee, and ankle) contractures determined by the passive range of motion (ROM). Contracture measurement will follow the CP Follow-up Program (CPUP) measurement manual, based on the guidelines of Norkin and White [42]. Measurements will be done using a universal goniometer and in standardized positions. Decreases in passive ROM are considered contractures if they fall within the red level of the “traffic light” system of the CPUP [42]. For the hip joint, abduction and extension ROM will be measured with the Ely test [43]. For the knee joint, extension along with popliteal angle will be measured [44]. Finally, for the ankle joint, dorsiflexion with knee flexed and extended along with plantarflexion will be measured. Assessors will be consistent across data collection points (screening, baseline, at 6 weeks, at 12 weeks, and at 24 weeks). Interrater reliability will be measured on 25% of the sample, chosen randomly to ensure consistency among assessors.

#### Randomization

Match or pair randomization will be used in this study to control for confounding effects of sex and GMFCS level. Children will be allocated to the MPH, modafinil, or placebo group using a concealed centralized electronic allocation application. Children will be matched for sex (male or female) and GMFCS (level I or II).

#### Sample Size

This study will include 12 children in each group (N=36). Due to the design of the study (ie, a pilot trial), power calculation will not be performed to estimate the sample size. Upon the completion of this study, our findings will provide effect sizes that are essential for future studies to conduct a power analysis.

#### Blinding Procedures

This study will be triple-masked (blinded), where treatment providers, outcome assessors, and participants (children and their legal guardians) will be unaware of the study group allocation. Two pharmacists and 1 study coordinator will be the only personnel exposed to the group allocation. The 2 pharmacists will carry out the randomization process and prepare the medication using a concealed nontransparent envelope and send it to the nursing staff, who will provide the treatment approximately 90 minutes before the physical therapy session. The study coordinator will track group allocation and deliver the prescriptions to the nursing staff before the administration of the drug. Physicians who prescribe MPH, modafinil, or placebo will use a customized sham form to ensure blinding. All primary and secondary outcomes will be collected by 2 physical therapists (PTs), who will be also blinded to the group allocation.

### Intervention

#### Medication

**Figure 2:**
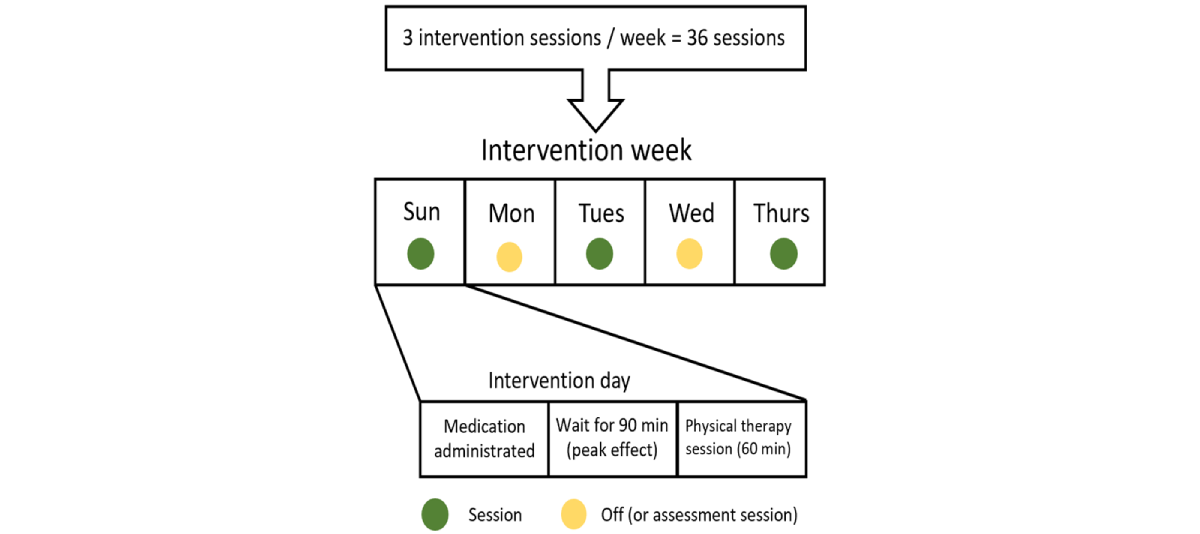
Illustration of the timing of the administration of the medication and physical therapy sessions.

Participants will receive 2 medications based on their group assignment. The MPH group will receive a quarter tablet of short-acting MPH (2.5 mg) and an oval-shaped placebo (100 mg) tablet that looks like a modafinil tablet. The modafinil group will receive a modafinil (100-mg) tablet and a circle-shaped quarter tablet of placebo that looks like an MPH tablet (2.5 mg). The placebo group (the active control) will receive 2 placebo tablets (a circle-shaped quarter tablet of MPH placebo and an oval-shaped tablet of modafinil placebo). All delivered medications (MPH, modafinil, and placebo) will be administered approximately 90 minutes before the physical therapy session, to use the peak medication effect to augment the outcomes of physical therapy [45]. The medication will be administered by a registered nurse and will be controlled by the pharmacy department at Jaber Al-Ahmed Hospital. The medication will be only prescribed on the day of a physical therapy session, which means that children will receive 3 medication doses per week over 12 consecutive weeks (the duration of the study). Since children need to receive the medication 90 minutes before their PT session, appointments will be scheduled on Sundays, Tuesdays, and Thursdays during the daytime (ie, from 8 AM to 1 PM; Figure 2). Any missed appointment will be documented, and the prescribed medication will be returned to the pharmacy. After 12 weeks of medication administration, participants will stop receiving the prescribed medication in this study, whether it was the active agent or placebo. A follow-up assessment session will be performed 12 weeks after the discontinuation of medication to examine the potential for long-lasting benefits from the received medication. Further analysis will be done to compare compliant versus noncompliant children.

All MPH (Ritalin, 10 mg; Novartis), modafinil (Provigil, 100 mg; Apotex), and placebo (circle-shaped MPH placebo, 10 mg; oval-shaped modafinil placebo, 100 mg; Kuwait Saudi Pharmaceutical Industries Co) bottles that will be used in this study will adhere to labeling requirements to ensure traceability and accountability, containing the following information: (1) the full name of the supplier of the medication and placebo bottles, (2) the batch number, and (3) the manufacturing and expiration dates. In addition, the medication and placebo tablets will be extracted from their original bottles and secured in individual containers with the aforementioned information and a patient identifier. The study coordinator is responsible for ensuring compliance with these labeling requirements, which will be recorded and will be made available for inspection upon request.

#### Physical Therapy Intervention

The physical therapy intervention will include therapeutic exercises that target different domains of the International Classification of Functioning, Health, and Disability (ICF), including body structure, activity, and participation. The dose of the physical therapy intervention will be as follows: (1) the frequency will be 3 sessions per week for 12 weeks; (2) the session time will be 60 minutes; (3) the type and intensity will be treatment activities that focus on joint ROM and muscle strength (body structure domain), locomotion training (body structure [endurance], activity [gait training], and participation [locomotive abilities] domains), reaching training (activity and participation domain), and motor control and motor learning training (activity and participation domain) (Table 1). A detailed manual will be provided to the treating PTs to ensure consistency in the treatment delivered to each child. After 12 weeks of receiving the physical therapy intervention, participants are entitled to enroll into any other rehabilitation program. At week 24, participants will complete an assessment session and provide information on whether they received rehabilitation after stopping the medication, if any, including duration of the session per day, frequency of sessions per week, and compliance to these sessions in the past 12 weeks.

**Table 1:** Detailed physical therapy plan.

ICF^a^ domain and type		Details
**Body structure and function**		
	Joint range of motion	Active and passive stretching (eg, squats and stepping up)
	Muscle strength	Active and resistive training (85% of 1-repetition maximum—build up to 3 sets of 10 repetitions)
**Activity**		
	Locomotion training	Treadmill walking—build up to 1.2 m/s with a 0.2 m/s increase per week or as tolerated Overground walking—over and around obstacles for 10 m or as tolerated
	Reaching training	Bimanual activities—reaching in all directions (with both hands or switching between hands)
	Postural control	Equilibrium reaction training— moving from a stable to an unstable surface with eyes open, then repeated with eyes closed
**Participation**		
	Motor control and learning	Training for activities of daily living—standing progression, walking progression, and higher function progression (stair climbing, running, and jumping)

^a^ICF: International Classification of Functioning, Health, and Disability.

#### Implementation of the Intervention

Data collection sessions (assessment data points) will be held on Sundays, Tuesdays, and Thursdays during the daytime (ie, 8 AM to 1 PM). Two PTs will be assigned to deliver physical therapy interventions throughout the study. Each PT will treat a range of 3 to 6 children per intervention period (n=36 intervention sessions). Two experienced investigators will supervise the physical therapy treatment and track the participants’ compliance. The expected time commitment for each child for each session is about 2 to 2.5 hours, with the first hour waiting for the medication’s peak effect and the remaining time for the physical therapy intervention.

### Outcomes

#### Primary Outcomes

After obtaining the consent and assent forms, caregivers will complete a questionnaire on demographic information, followed by a body weight and height assessment of each child. The primary outcomes of this study will be the GMFM-66 and MAS scores for the LEs and UEs to assess changes in functional motor performance and spasticity, respectively. The assessment session will be done by a trained PT who is different from the PTs that will deliver the intervention. There will be 4 examination sessions (baseline, at 6 weeks, at 12 weeks, and at 24 weeks) that will last approximately 2 hours—for both primary and secondary outcomes. These assessment sessions will be scheduled separately from the intervention sessions. The GMFM-66 is designed to examine gross motor activities in five domains: (1) lying and rolling, (2) sitting, (3) crawling and kneeling, (4) standing, and (5) walking, running, and jumping [46]. The GMFM-66 scores range between 0 to 3, where 0 corresponds to “does not initiate,” 1 corresponds to “initiates,” 2 corresponds to “partially completes,” and 3 corresponds to “completes” [46]. Items that a child refuses to perform or that are not administered are scored as “not tested” [46]. Scoring will be done using the Gross Motor Ability Estimator (GMAE), where each score is entered and then converted into an internal level to calculate the total score for each child’s gross motor function. The GMFM-66 is a valid and reliable tool to assess gross motor function in children with CP [47]. In addition, we will use the MAS for the LE (hip extensors and adductors and ankle plantar flexors) and UE (shoulder internal rotators and flexors and elbow flexors) musculature as the second primary outcome. The MAS scores range from 0 (no increase in resistance to stretch) to 4 (complete rigidity) [48]. Children will lay supine on a flat bed with their head at midline and the arm beside the trunk [48]. Each extremity will be examined separately. The examiner will rapidly move the child’s extremity throughout the range within 1 second [48]. The examiner will determine the MAS score after applying 3 passive movements [48]. This MAS has been shown to have a good intrarater reliability for the LEs (intraclass correlation [ICC]=0.644; Cohen κ=0.488) [49]. The minimum clinically important differences (MCIDs) for the GMFM-66 are 1.7 and 1.0 for GMFCS levels I and II, respectively [50].

#### Secondary Outcomes

The secondary outcomes will include the Timed Up and Go (TUG) test, 5 Times Sit to Stand (5XSTS) test, Modified Clinical Test for Sensory Interaction of Balance (MCTSIB), and 10-Meter Walk Test (10MWT). For the TUG, participants will stand up from a 45-cm height armchair, walk at their typical, comfortable pace around a cone that will be placed 3 meters away from the chair, then walk back to the chair and sit down. Time(s) to complete the task will be recorded as the score of this test using a stopwatch. The TUG has excellent test-retest reliability in children with CP (ICC=0.91-0.99) and MCIDs of 0.36 and 0.87 for GMFCS levels I and II, respectively (ie, a large effect size) [51].

For the 5XSTS, participants will stand up from a 45-cm armchair and sit down as fast as they can. Time(s) will be used for scoring this test. This test has excellent test-retest reliability in children with CP (ICC=0.91) [52]. Of note, MCID data are not available for children with CP; however, the minimal detectable change for this test is 0.06 seconds [53].

The MCTSIB measures balance in 4 conditions, measured for a maximum of 3 trials of 30 seconds for each condition: firm floor with eyes open, then closed; and foam (unstable surface) with eyes open, then closed [54]. This test has excellent test-retest reliability (ICC=0.91) [54].

For the 10MWT, participants will be instructed to walk at their comfortable pace for 10 meters; time(s) will be recorded as the score of this test. This test is to measure gait speed and variability and has fair test-retest reliability (ICC=0.81) [55].

### Statistical Analysis

SPSS (version 29; IBM Corp) will be used to conduct all the data analyses in this study. For changes in the primary outcomes, we will use a repeated-measures ANOVA to examine changes in the GMFM-66 between the 3 intervention groups across all testing time points (ie, baseline, at 6 weeks, at 12 weeks, and at 24 weeks). To examine changes in MAS between the 3 intervention groups, we will use Kruskal-Wallis *H* test because MAS is an ordinal scale. Finally, we will use multivariate linear mixed models to determine changes in the associated secondary outcomes between the 3 intervention groups.

### Adverse Events

At the beginning of each intervention week, a questionnaire will be administered by the PTs inquiring about adverse effects. Any adverse events will be addressed immediately by the research team. Any potential risks of using CNS medications will be recorded in the informed consent and assent forms and will be explained to the caregiver of each child. In case of an adverse event, we will refer the participant directly to the emergency department to receive medical care and record the incident of the adverse event. Additionally, we will notify the institutional review board office at Kuwait University and the Ministry of Health of Kuwait about any potential incidents. Finally, should we encounter 2 or more incidents per medication group, we will stop the intervention for that specific group and document it in our records.

### Data Collection and Management

Data collection will be performed at baseline (before randomization), 6 weeks, and 12 weeks, as well as at 24 weeks (as a test of long-lasting effects) after starting the intervention. A data monitoring committee (DMC) will be assigned that will include a research assistant and a pharmacist. Members of the DMC will be exposed to the randomization process and will manage adverse events throughout the protocol. Compliance to medication and physical therapy will be collected every session by the PTs, using an online Google form; Microsoft Excel sheets and participant ID numbers will be used to identify each participant. Only the DMC members will have access to this sheet, in which no identifiable information of participants will be included.

### Confidentiality

All identifiable data of participants will be protected and decoded in the data collection sheet. After agreement to participate, signed consent and assent forms will be stored in a locked locker at the principal investigator’s office. All other study documents (eg, data collection sheet, randomization and mediation prescriptions, and demographic data questionnaires) will use a participant ID number that will replace the name of the child.

### Ancillary and Posttrial Care

Due to the pilot nature of this study, the results will not provide definite findings regarding the effectiveness of CNS stimulant medications in reducing spasticity and improving motor function in children with CP. Therefore, we are unable to recommend the prescribing of any CNS medication for children with CP until the findings of advanced phases of this project indicate their efficacy and effectiveness.

## Results

The protocol has been accepted by Kuwait University (VDR/EC-225) and the Ministry of Health of Kuwait (2022/2157). The inclusion of participants will start in June 2024. The findings of this study are intended to be disseminated at several scientific meetings, including the American Academy of Cerebral Palsy and Developmental Medicine and the AAN annual meetings. Additionally, the findings of this study will be submitted for publication to scientific journals specialized in the field of CP and developmental medicine.

## Discussion

### Principal Findings

This study protocol details the background and design of a pilot RCT to determine changes in motor performance and spasticity as a result of the use of CNS stimulant medications in children with CP. We expect the findings of this study to document the safety and tolerability of using these medications in children with CP. We also expect that there will be a trend of improvements in the GMFM-66 and MAS in the CNS stimulant groups (ie, MPH and modafinil) compared to the placebo group. We anticipate that these changes will impact quality of life and the overall function of children with CP, demonstrated by improvement trends in secondary outcomes, where these improvements could document positive effects of CNS stimulants in improving the health of patients with CP.

The findings of this study will be shared and disseminated through multiple scientific mediums, including annual conferences and scientific meetings, as well as peer-reviewed scientific journals. The functional and motor improvements that could result from using CNS stimulants in this population could reduce the consequences of impairments associated with CP. Therefore, using CNS stimulants could help in reducing the financial burden associated with treating CP functional impairments on the health care system.

### Limitations

The complexity of CP and the heterogeneity of motor impairments may contribute to variations in response to medication in this study. However, we limited participation in this study to participants with GMFCS levels I and II to minimize the effect of severe motor impairments. Although not an issue in pilot studies, dropout across the study groups may occur. Nevertheless, this concern could be mitigated by providing physical therapy sessions to all groups in addition to medications. Regardless, CP motor treatment is valued across parents of children with CP and could help in reducing attrition. Finally, information obtained from the physical therapy intervention will be important for revising the conceptual model for the intervention and will inform the next step of the trial.

### Conclusions

Evidence suggests promising results of using CNS stimulant medications for reducing spasticity and improving physical function in children with spastic CP. The combination of CNS stimulant medications and controlling for rehabilitation has not been studied yet. The findings of this study may suggest positive effects of using CNS stimulant medications for reducing spasticity and improving physical function in children with spastic CP.

## Abbreviations

10MWT: 10-Meter Walk Test
5XSTS: 5 Times Sit to Stand
AAN: American Academy of Neurology
ADHD: attention-deficit/hyperactivity disorder
ADL: activity of daily living
BoNT-A: botulinum toxin type A
CONSORT: Consolidated Standards of Reporting Trials
CP: cerebral palsy
CPUP: cerebral palsy follow-up program
CNS: central nervous system
GABA: gamma-amino butyric acid
GMAE: Gross Motor Ability Estimator
GMFCS: Gross Motor Function Classification System
GMFM: Gross Motor Function Measure
ICC: intraclass correlation
ICF: International Classification of Functioning, Health, and Disability
LE: lower extremity
MAS: Modified Ashworth Scale
MCID: minimum clinically important difference
MCTSIB: Modified Clinical Test for Sensory Interaction of Balance
MPH: methylphenidate
PT: physical therapist
RCT: randomized controlled trial
ROM: range of motion
SPIRIT: Standard Protocol Items: Recommendation for Interventional Trials
TUG: Timed Up and Go
UE: upper extremity

## Data Availability

Data sharing is not applicable to this article as no data sets were generated or analyzed during this study.
